# Integration of HIV in child survival platforms: a novel programmatic pathway towards the 90–90–90 targets

**DOI:** 10.7448/IAS.18.7.20250

**Published:** 2015-12-02

**Authors:** Dick D Chamla, Shaffiq Essajee, Mark Young, Scott Kellerman, Ronnie Lovich, Nandita Sugandhi, Anouk Amzel, Chewe Luo

**Affiliations:** 1 Health Section, UNICEF, New York, NY, USA; 2 HIV Department, World Health Organization, Geneva, Switzerland; 3 Global HIV Program, Management Sciences for Health, Arlington, VA, USA; 4 MCH Department, Elizabeth Glaser Pediatric AIDS Foundation, Washington, DC, USA; 5 HIV Department, Clinton Health Access Initiative, New York, NY, USA; 6 HIV Office, USAID, Washington, DC, USA; 7 HIV Section, UNICEF, New York, NY, USA

**Keywords:** integration, HIV, child survival, double dividend, maternal-child health, PITC

## Abstract

**Introduction:**

Integration of HIV into child survival platforms is an evolving territory with multiple connotations. Most literature on integration of HIV into other health services focuses on adults; however promising practices for children are emerging. These include the Double Dividend (DD) framework, a new programming approach with dual goal of improving paediatric HIV care and child survival. In this commentary, the authors discuss why integrating HIV testing, treatment and care into child survival platforms is important, as well as its potential to advance progress towards global targets that call for, by 2020, 90% of children living with HIV to know their status, 90% of those diagnosed to be on treatment and 90% of those on treatment to be virally suppressed (90–90–90).

**Discussion:**

Integration is critical in improving health outcomes and efficiency gains. In children, integration of HIV in programmes such as immunization and nutrition has been associated with an increased uptake of HIV infant testing. Integration is increasingly recognized as a case-finding strategy for children missed from prevention of mother-to-child transmission programmes and as a platform for diffusing emerging technologies such as point-of-care diagnostics. These support progress towards the 90–90–90 targets by providing a pathway for early identification of HIV-infected children with co-morbidities, prompt initiation of treatment and improved survival. There are various promising practices that have demonstrated HIV outcomes; however, few have documented the benefits of integration on child survival interventions. The DD framework is well positioned to address the bidirectional impacts for both programmes.

**Conclusions:**

Integration provides an important programmatic pathway for accelerated progress towards the 90–90–90 targets. Despite this encouraging information, there are still challenges to be addressed in order to maximize the benefits of integration.

## Introduction

Integration of health services for children that incorporate aspects of HIV care and treatment with other childhood illnesses is high on the global health and development agenda. Yet defining and measuring integration remains elusive. In the health sector, integration of services in primary health care (PHC) can be traced back to the Alma Ata declaration adopted in 1978, which recognized the importance of primary health care as key to achieving the goal of “Health For All” [1]. The Paris Declaration on Aid Effectiveness in 2005 further reaffirmed the importance of integration as a country-owned process built upon existing programmes [2]. For decades, debates on the comparative impact of *vertical* versus *integration* systems have had polarizing views mainly due to lack of hard evidence [3].

The World Health Organization defines *integration* as the management and delivery of health services, where clients receive a continuum of preventive and curative services according to their needs over time and across different levels of the health system [4]. In practice, integration has many forms, including co-location of services at one facility, co-delivery of multiple services by one programme or combined services for every client in one encounter to ensure multiple needs are met. More often, the idea of comprehensiveness overlaps with that of integration [5]. There is also a range of descriptions of integration, such as a “supermarket” approach for service delivery, “one-stop shop” or delivery of services “under the same roof.” A more recent concept that has emerged in paediatric HIV is that of “smart integration,” which underlines the importance of data-driven, targeted and feasible choices in models of integrated services aimed at maximizing child survival outcomes. Smart integration calls for rigorous analysis of gaps in service delivery and coverage, to guide the design of feasible and targeted integration of services. Additionally, it calls for robust monitoring strategies to be in place to track outcomes and potential negative consequences for both new and existing services. In the HIV field, there is growing literature on improved outcomes following integration of HIV with programmes such as tuberculosis (TB) and family planning among adult populations [6–8]; however, such evidence among children remains rudimentary.

This paper examines why integrating HIV services into child survival platforms is important and discusses promising strategies, opportunities and challenges of integrating HIV services into selected child survival programmes to improve both HIV and child survival outcomes. This includes the Double Dividend (DD) framework, a new programming approach that describes the bidirectional benefits of integrating HIV services with child survival platforms [9]. The article aims to show that integration is a key strategy needed for survival of children living with HIV and for the delivery of services and uptake of new technologies critical to reaching the new global target that calls for, by 2020, 90% of children living with HIV to know their status; 90% of those diagnosed to be on treatment; and 90% of those on treatment to be virally suppressed (90–90–90).

## Discussion

The rationale for integration dwells on its potential to improve service delivery, health outcomes and efficiencies [10]. For example, in Uganda, the introduction of Integrated Management of Childhood Illness (IMCI) led to improved performance of trained health workers and superior quality of care delivered to children aged under five years [11]. Similar findings on improved outcomes following IMCI implementation were reported in Bangladesh, South Africa and Tanzania [12–14]. A newborn mortality reduction of 34 to 62% was also demonstrated through the delivery of a package of interventions shortly after birth [15,16]. For HIV-exposed children, there are indications of improved uptake of HIV testing following integration with programmes such as immunization and nutrition [17–19]. However, there is a lack of evidence of potential impact on child survival programmes following their integration with HIV.

Integration of HIV testing in child survival programmes is increasingly recognized as a case-finding strategy for children who were missed in prevention of mother-to-child transmission of HIV (PMTCT) programmes or who were infected late during the breastfeeding period. This issue is crucial, as global coverage of early infant diagnosis remains low at 39% among the 22 priority countries that contribute to over 90% of new paediatric HIV infections globally [20]. In spite of growing literature on HIV co-morbidities [21,22], the contribution of HIV in major causes of under-five mortality rates (U5MR), such as pneumonia or diarrhoea, remains largely unknown. Given the potential for case finding within service delivery platforms for sick, malnourished and well-child care, HIV testing at various child survival entry points offers significant potential for accelerated case finding, clear pathways to HIV treatment initiation and may contribute to retention in care. However, decades of vertical programming of paediatric HIV care has missed these opportunities, resulting in poor survival outcomes for children with co-morbidities [22].

Recent successes of the PMTCT programmes in reducing HIV transmission in the prenatal and postnatal periods provide another reason for integration of services for children. There are fewer HIV-infected children being born and identified through successful PMTCT programmes [23]. Therefore, the majority of yet-unidentified HIV-infected children are those born to women that were missed or lost from PMTCT programmes. Most of these unidentified HIV-infected children could be identified through HIV testing in other facility-based programmes that care for children, such as immunization, nutrition or inpatient services. Last, the importance of integration could also be viewed as a platform for diffusing technological innovations such as point-of-care diagnostics (PoC) for viral load assays [24] in order to maximize their impacts in child survival programmes.

There are two approaches for integrating HIV testing into child survival platforms that can be considered: general integrated platforms, such as in immunization sites, where integration would enable universal HIV testing or screening of all children, versus targeted integration, where the focus for testing would be on a subset of high risk children (such as sick or malnourished children). The choice of these approaches is likely determined by each country's HIV epidemic context and health system characteristics. For instance, integration of HIV testing into routine immunization sessions may be ideal in high HIV prevalence countries [25], whereas screening of all infants for risk of HIV exposure may be a more effective practice in low level epidemics.

Beyond integration of HIV testing, there is a need to establish feasible approaches to improving retention of HIV-exposed infants and HIV-infected infants and children in HIV care and treatment services within maternal and child health (MCH) settings. What is seen in the literature is that most of the focus of integration is on HIV testing and it does not examine delivery of paediatric HIV treatment through MCH service delivery points. In Mozambique, care of HIV-exposed and -infected children is being delivered together with care for other at-risk children through “Child at Risk” clinics providing an integrated model of service delivery for all paediatric health issues.

### Promising integrated approaches

Many countries have integrated HIV testing into one or more child survival platforms; however few have documented the specific outcomes due to lack of robust evaluation designs. There are a few instances, such as integration of HIV testing in immunization and nutrition programmes, paediatric inpatient units and community or home-based programmes [26–36], that have demonstrated excellent outcomes in HIV case finding (Box 1). Fewer studies, however, have shown the effect on child health platforms when HIV testing is incorporated. The need for maximizing the outcomes for both child survival and paediatric HIV interventions has been the guiding mantra for the DD framework.

**Box 1. Promising approaches for integrating HIV into child survival platforms**

HIV and immunization integration

Increased uptake of HIV testing through immunization services has been well described [18]. In some settings, more than 90% of mothers accepted the offer to test their infants during immunization sessions [26]. Other studies showed a sevenfold greater proportion of infants receiving HIV testing in immunization clinics than in under-five clinics [19]. There is growing consensus on the effectiveness of this approach in increasing coverage of HIV infant testing and thereby helping to reach the UNAIDS target of 90% of infected children knowing their HIV status. However, further study of the effects of that integration on basic immunization platforms as well as the yield from case findings in different epidemic contexts would be valuable. There remains the challenge of stigma reported by other studies [29], which needs careful attention during design and monitoring of new integration initiatives.

HIV and nutrition integration

Malnourished children infected with HIV have an increased risk of mortality [22]. Routine HIV testing for early identification and prompt initiation of antiretroviral therapy (ART) have been associated with improved nutritional recovery and overall survival of infected children [17]. Uptake of HIV testing of more than 94% for children in nutrition programmes has been reported [30]. As such, both facility- and community-based nutrition therapeutic centres have been valuable entry points for HIV testing in most countries [30]. There are now studies confirming the association between food insecurity and unsuppressed viral load among ART patients [27,45].

HIV testing in children’s clinics and wards

There is mounting experience in providing HIV testing to hospitalized or out-patient paediatric populations in high HIV prevalence settings. In Zambia over 87% of paediatric patients admitted to one hospital received HIV testing [28]. Of those, 29% were identified as HIV positive. In one meta-analysis, up to one-fifth of pneumonia cases and 60% of pneumonia deaths were shown to occur in HIV-infected children [21]. As the efficiency and reach of prevention of mother-to-child transmission of HIV (PMTCT) programmes increase, a focus on children sick enough to be admitted to hospital may prove effective in early identification of HIV-infected children that “fall through the cracks” of PMTCT services.

HIV integration in community-based programmes

For decades, a community component of paediatric HIV response has been overlooked. Promising community-based paediatric HIV practices are now evident. The “Tingathe” community project in Malawi successfully used community health workers, with increased uptake of maternal and infant HIV testing [32]. Home-based HIV testing for children has been piloted in countries such as Swaziland [36]. In 2013, UNICEF and WHO revised their community health care training packages and materials for integrated community case management for pneumonia, diarrhoea and malaria, caring for newborns in the community and care of the well child to include HIV and tuberculosis [31]. Upcoming pilot implementation of these adapted materials remains critical.

### Double Dividend

The DD is a framework intended to catalyze actions toward the dual goals of accelerating paediatric HIV prevention, care and treatment, while contributing to improvements in child survival [9]. It calls for a systematic approach from both a child health and paediatric HIV perspective, to strengthen linkages and targeted integration across the health system based on analysis of gaps in coverage and needs for strengthening service delivery platforms. It also acknowledges the need to improve the response for HIV-exposed children, who face additional vulnerabilities as the risk of death among HIV-exposed uninfected children continues to rise [37]. However most programmes have yet to incorporate this emerging evidence in their HIV response.

Through preliminary analysis undertaken during a think tank exercise in Harare, the architects of DD identified five key areas where integration of HIV and child survival platforms can be mutually beneficial and therefore improve child outcomes – in postnatal care settings, immunization clinics, nutrition points, IMCI and integrated community case management for pneumonia, diarrhoea and malaria. The DD framework proposes four operational steps that will enable countries to 1) identify major causes of U5MR, paediatric HIV infections and the unmet care needs in these areas; 2) design integrated approaches for programming in child survival and paediatric HIV based on the needs assessment; 3) deliver and monitor the package of integrated services developed; and 4) work to ensure sustainability.

There are several novel dimensions to the framework: an expanded definition of the paediatric HIV-affected population to include HIV-exposed uninfected children; targeted integration of paediatric HIV in broader maternal, newborn and child health (MNCH) platforms; and greater understanding of the role that paediatric HIV infection plays in relation to common childhood diseases such as pneumonia, malaria and diarrhoea. The DD calls for a major shift in paediatric HIV programming, away from its current vertical delivery model, with the overall aim of identifying areas where investments of effort and resources will maximize prevention, care and treatment outcomes while contributing to strengthened child survival platforms.

The DD comes during dialogues of post-millennium development goals and new calls to action for child and newborn health, with new targets and timelines for achieving goals [38]. New global targets for paediatric HIV, 90–90–90, have also been endorsed [39]. Convergence of these initiatives to achieve the 90–90–90 targets in countries should be an obvious next step. Among the 11 countries endorsing the DD in 2013, Zimbabwe has begun taking steps to ensure that accelerated action to find HIV-infected children and initiate treatment can be undertaken through strengthened child survival platforms. There are critical lessons that can be adapted in other countries.

### Opportunities

As the world moves towards sustainable development goals, we have an unprecedented opportunity to reduce the silos existing in the implementation of paediatric HIV programmes. In 2011, in the United Nations General Assembly High Level Meeting on HIV, heads of state committed to eliminating parallel systems and integrating HIV services into health and broader development programming. Efforts are needed to ensure HIV-infected children are included in the Universal Health Coverage Initiative [40], health system strengthening and equity agendas and when addressing unmet needs for newborn and child survival [38]. The momentum for childhood TB efforts also provides an important opportunity for child survival to be further optimized [41]. Other opportunities for inclusion of HIV into child survival platforms include its integration in the already scaled-up PMTCT/antiretroviral therapy (ART) programming for women in MCH settings (Option B+) [42,43]. Likewise, the community health worker (CHW) movements such as One Million CHW by 2015 [44] have been silent on the unmet needs in paediatric HIV – missing a clear opportunity to improve survival of children exposed to or infected with HIV.

### Challenges for integration

The majority of critical gaps that hamper HIV service delivery also threaten basic health services. In many countries, MNCH, particularly post-natal platforms, is underdeveloped, under-utilized and in most cases under-funded. The extent and monitoring of outcomes following integration remains a challenge. For instance, most literature has focused on integration of HIV and child survival platforms at the service delivery level (Box 1), with little information on full integration across the critical elements of health systems such as governance, human resources, information or financing. As previously discussed, the outcomes on child survival platforms following their integration with HIV are rarely reported. For example, understanding how HIV integration into vaccination sites may impact completion of the full immunization schedule could be beneficial to the immunization community. As the impact of integration of HIV services into child health platforms is not yet widely known, robust studies are needed to evaluate the effects.

### Integration and the 90–90–90 goals

Addressing persistent gaps in the provision of paediatric HIV services and ultimately the global targets of 90–90–90 by 2020 requires innovative approaches. Integration, as one of the programmatic innovations, has the potential to accelerate this goal through intensified case finding in child survival platforms, timely treatment initiation, improved retention and ultimately long-term survival of children exposed to or infected with HIV. Implementing the approaches that integrate HIV testing into child survival platforms (as presented in Box 1), would greatly accelerate progress towards the first 90 goal – 90% of HIV-infected children knowing their status. Delivery of paediatric ART in the MCH facilities alongside PMTCT programming (Option B+) remains a critical strategy to advance the second 90 goal – ensuring that 90% of those who test positive receive treatment. Synergies between HIV and other programmes such as nutrition enhance viral suppression for those on ART [27,45] and improve the achievement of the third 90 goal – 90% of children on ART to maintain viral suppression. These benefits are further amplified by the potential for efficiency gains and dissemination of new technologies necessary to accelerate progress towards the 90–90–90 goals. Strategies such as DD provide new momentum for programming approaches to reach the 90–90–90 goals. There are, however, challenges that need to be addressed and joint investments are needed to ensure that integration achieves maximum impacts for both platforms.

## Conclusions

The field of integration of HIV into child survival platforms is evolving with remarkable prospects. Its potential role in case identification, treatment initiation and dissemination of new technologies should form a critical part of strategies towards the achievement of the 90–90–90 goals. The potential for the field of integration could be additionally enhanced by combining with emerging technological advances such as PoC diagnostics and optimized paediatric antiretroviral drug formulations. Defining the bidirectional dividends, including the burden of HIV in other major child health diseases, will enhance optimal care and joint investments. Adequate investments and implementation research are needed to ensure that optimal integrated models are identified and scaled up, both to achieve the goals of 90–90–90 in paediatric HIV and to improve child survival outcomes.
